# Revolutionizing healthcare: the role of artificial intelligence in clinical practice

**DOI:** 10.1186/s12909-023-04698-z

**Published:** 2023-09-22

**Authors:** Shuroug A. Alowais, Sahar S. Alghamdi, Nada Alsuhebany, Tariq Alqahtani, Abdulrahman I. Alshaya, Sumaya N. Almohareb, Atheer Aldairem, Mohammed Alrashed, Khalid Bin Saleh, Hisham A. Badreldin, Majed S. Al Yami, Shmeylan Al Harbi, Abdulkareem M. Albekairy

**Affiliations:** 1 Department of Pharmacy Practice, College of Pharmacy, King Saud bin Abdulaziz University for Health Sciences, Prince Mutib Ibn Abdullah Ibn Abdulaziz Rd, Riyadh, 14611 Saudi Arabia; 2 King Abdullah International Medical Research Center, Riyadh, Saudi Arabia; 3 Pharmaceutical Care Department, King Abdulaziz Medical City, National Guard Health Affairs, Riyadh, Saudi Arabia; 4 Department of Pharmaceutical Sciences, College of Pharmacy, King Saud bin Abdulaziz University for Health Sciences, Riyadh, Saudi Arabia

**Keywords:** AI, Healthcare, Patient care, Quality of life, Clinicians, Decision-making, Personalized treatment plans

## Abstract

**Introduction:**

Healthcare systems are complex and challenging for all stakeholders, but artificial intelligence (AI) has transformed various fields, including healthcare, with the potential to improve patient care and quality of life. Rapid AI advancements can revolutionize healthcare by integrating it into clinical practice. Reporting AI’s role in clinical practice is crucial for successful implementation by equipping healthcare providers with essential knowledge and tools.

**Research Significance:**

This review article provides a comprehensive and up-to-date overview of the current state of AI in clinical practice, including its potential applications in disease diagnosis, treatment recommendations, and patient engagement. It also discusses the associated challenges, covering ethical and legal considerations and the need for human expertise. By doing so, it enhances understanding of AI’s significance in healthcare and supports healthcare organizations in effectively adopting AI technologies.

**Materials and Methods:**

The current investigation analyzed the use of AI in the healthcare system with a comprehensive review of relevant indexed literature, such as PubMed/Medline, Scopus, and EMBASE, with no time constraints but limited to articles published in English. The focused question explores the impact of applying AI in healthcare settings and the potential outcomes of this application.

**Results:**

Integrating AI into healthcare holds excellent potential for improving disease diagnosis, treatment selection, and clinical laboratory testing. AI tools can leverage large datasets and identify patterns to surpass human performance in several healthcare aspects. AI offers increased accuracy, reduced costs, and time savings while minimizing human errors. It can revolutionize personalized medicine, optimize medication dosages, enhance population health management, establish guidelines, provide virtual health assistants, support mental health care, improve patient education, and influence patient-physician trust.

**Conclusion:**

AI can be used to diagnose diseases, develop personalized treatment plans, and assist clinicians with decision-making. Rather than simply automating tasks, AI is about developing technologies that can enhance patient care across healthcare settings. However, challenges related to data privacy, bias, and the need for human expertise must be addressed for the responsible and effective implementation of AI in healthcare.

## Introduction

Artificial Intelligence (AI) is a rapidly evolving field of computer science that aims to create machines that can perform tasks that typically require human intelligence. AI includes various techniques such as machine learning (ML), deep learning (DL), and natural language processing (NLP). Large Language Models (LLMs) are a type of AI algorithm that uses deep learning techniques and massively large data sets to understand, summarize, generate, and predict new text-based content [1–3]. LLMs have been architected to generate text-based content and possess broad applicability for various NLP tasks, including text generation, translation, content summary, rewriting, classification, categorization, and sentiment analysis. NLP is a subfield of AI that focuses on the interaction between computers and humans through natural language, including understanding, interpreting, and generating human language. NLP involves various techniques such as text mining, sentiment analysis, speech recognition, and machine translation. Over the years, AI has undergone significant transformations, from the early days of rule-based systems to the current era of ML and deep learning algorithms [1–3].

AI has evolved since the first AI program was developed in 1951 by Christopher Strachey. At that time, AI was in its infancy and was primarily an academic research topic. In 1956, John McCarthy organized the Dartmouth Conference, where he coined the term “Artificial Intelligence.“ This event marked the beginning of the modern AI era. In the 1960 and 1970 s, AI research focused on rule-based and expert systems. However, this approach was limited by the need for more computing power and data [4].

**Fig. 1:**
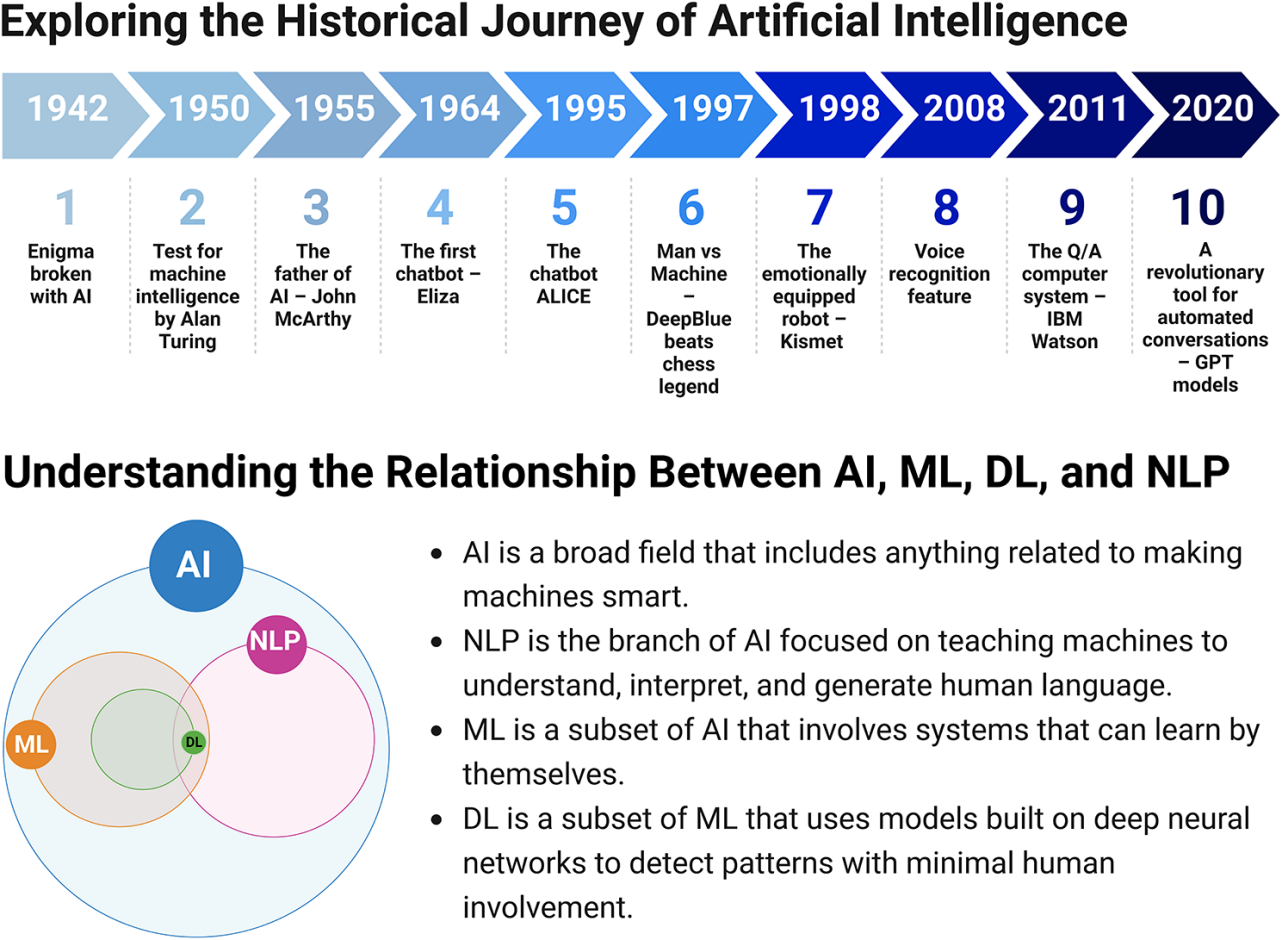
Tracing the Evolution of AI with a Better Understanding of the Relationship Between AI, ML, DL, and NLP

In the 1980 and 1990 s, AI research shifted to ML and neural networks, which allowed machines to learn from data and improve their performance over time. This period saw the development of systems such as IBM’s Deep Blue, which defeated world chess champion Garry Kasparov in 1997. In the 2000s, AI research continued to evolve, focusing on NLP and computer vision, which led to the development of virtual assistants, such as Apple’s Siri and Amazon’s Alexa, which could understand natural language and respond to user requests (Fig. 1) [3, 4].

Today, AI is transforming healthcare, finance, and transportation, among other fields, and its impact is only set to grow. In academia, AI has been used to develop intelligent tutoring systems, which are computer programs that can adapt to the needs of individual students. These systems have improved student learning outcomes in various subjects, including math and science. In research, AI has been used to analyze large datasets and identify patterns that would be difficult for humans to detect; this has led to breakthroughs in fields such as genomics and drug discovery. AI has been used in healthcare settings to develop diagnostic tools and personalized treatment plans. As AI continues to evolve, it is crucial to ensure that it is developed responsibly and for the benefit of all [5–8].

The rapid progression of AI technology presents an opportunity for its application in clinical practice, potentially revolutionizing healthcare services. It is imperative to document and disseminate information regarding AI’s role in clinical practice, to equip healthcare providers with the knowledge and tools necessary for effective implementation in patient care. This review article aims to explore the current state of AI in healthcare, its potential benefits, limitations, and challenges, and to provide insights into its future development. By doing so, this review aims to contribute to a better understanding of AI’s role in healthcare and facilitate its integration into clinical practice.

## Materials and methods

### Search strategy and inclusion

Indexed databases, including PubMed/Medline (National Library of Medicine), Scopus, and EMBASE, were independently searched with notime restrictions, but the searches were limited to the English language.

### Databases search protocol and keywords

In the review article, the authors extensively examined the use of AI in healthcare settings. The authors analyzed various combinations of keywords such as NLP in healthcare, ML in healthcare, DL in healthcare, LLM in healthcare, AI in personalized medicine, AI in patient monitoring, AI ethics in healthcare, predictive analytics in healthcare, AI in medical diagnosis, and AI applications in healthcare. By imposing language restrictions, the authors ensured a comprehensive analysis of the topic.

### Data extraction

Publications were screened through a meticulous review of titles and abstracts. Only those that met the specific criteria were included. Any disagreements or concerns about the literature or methodology were discussed in detail among the authors.

## AI assistance in diagnostics

### Diagnosis accuracy

With all the advances in medicine, effective disease diagnosis is still considered a challenge on a global scale. The development of early diagnostic tools is an ongoing challenge due to the complexity of the various disease mechanisms and the underlying symptoms. AI can revolutionize different aspects of health care, including diagnosis. ML is an area of AI that uses data as an input resource in which the accuracy is highly dependent on the quantity as well as the quality of the input data that can combat some of the challenges and complexity of diagnosis [9]. ML, in short, can assist in decision-making, manage workflow, and automate tasks in a timely and cost-effective manner. Also, deep learning added layers utilizing Convolutional Neural Networks (CNN) and data mining techniques that help identify data patterns. These are highly applicable in identifying key disease detection patterns among big datasets. These tools are highly applicable in healthcare systems for diagnosing, predicting, or classifying diseases [10].

AI is still in its early stages of being fully utilized for medical diagnosis. However, more data are emerging for the application of AI in diagnosing different diseases, such as cancer. A study was published in the UK where authors input a large dataset of mammograms into an AI system for breast cancer diagnosis. This study showed that utilizing an AI system to interpret mammograms had an absolute reduction in false positives and false negatives by 5.7% and 9.4%, respectively [11]. Another study was conducted in South Korea, where authors compared AI diagnoses of breast cancer versus radiologists. The AI-utilized diagnosis was more sensitive to diagnose breast cancer with mass compared to radiologists, 90% vs. 78%, respectively. Also, AI was better at detecting early breast cancer (91%) than radiologists 74% [12].

Furthermore, a study utilized deep learning to detect skin cancer which showed that an AI using CNN accurately diagnosed melanoma cases compared to dermatologists and recommended treatment options [13, 14]. Researchers utilized AI technology in many other disease states, such as detecting diabetic retinopathy [15] and EKG abnormality and predicting risk factors for cardiovascular diseases [16, 17]. Furthermore, deep learning algorithms are used to detect pneumonia from chest radiography with sensitivity and specificity of 96% and 64% compared to radiologists 50% and 73%, respectively [18]. Also, a study was done on a dataset of 625 cases to diagnose acute appendicitis early to predict the need for appendix surgery using various ML techniques; the results showed that the random forest algorithm achieved the highest performance, accurately predicting appendicitis in 83.75% of cases, with a precision of 84.11%, sensitivity of 81.08%, and specificity of 81.01%. The improved method aids healthcare specialists in making informed decisions for appendicitis diagnoses and treatment. Furthermore, the authors suggest that similar techniques can be utilized to analyze images of patients with appendicitis or even to detect infections such as COVID-19 using blood specimens or images [19].

AI tools can improve accuracy, reduce costs, and save time compared to traditional diagnostic methods. Additionally, AI can reduce the risk of human errors and provide more accurate results in less time. In the future, AI technology could be used to support medical decisions by providing clinicians with real-time assistance and insights. Researchers continue exploring ways to use AI in medical diagnosis and treatment, such as analyzing medical images, X-rays, CT scans, and MRIs. By leveraging ML techniques, AI can also help identify abnormalities, detect fractures, tumors, or other conditions, and provide quantitative measurements for faster and more accurate medical diagnosis.

Clinical laboratory testing provides critical information for diagnosing, treating, and monitoring diseases. It is an essential part of modern healthcare which continuously incorporates new technology to support clinical decision-making and patient safety [20]. AI has the potential to transform clinical laboratory testing by improving the accuracy, speed, and efficiency of laboratory processes. The role of AI in clinical microbiology is currently progressing and expanding. Several ML systems were developed to detect, identify, and quantify microorganisms, diagnose and classify diseases, and predict clinical outcomes. These ML systems used data from various sources to build the AI diagnosis such as genomic data of microorganisms, gene sequencing, metagenomic sequencing results of the original specimen, and microscopic imaging [21]. Moreover, gram stain classification to gram positives/negatives and cocci/rods is another essential application of using deep convolutional neural networks that reveal high sensitivity and specificity [22]. A published systematic review showed that numerous MLs were evaluated for microorganism identification and antibiotic susceptibility testing; however, several limitations are associated with the current models that must be addressed before incorporating them into clinical practice [23]. For malaria, Taesik et al. found that using ML algorithms combined with digital in-line holographic microscopy (DIHM) effectively detected malaria-infected red blood cells without staining. This AI technology is rapid, sensitive, and cost-effective in diagnosing malaria [24].

The projected benefits of using AI in clinical laboratories include but are not limited to, increased efficacy and precision. Automated techniques in blood cultures, susceptibility testing, and molecular platforms have become standard in numerous laboratories globally, contributing significantly to laboratory efficiency [21, 25]. Automation and AI have substantially improved laboratory efficiency in areas like blood cultures, susceptibility testing, and molecular platforms. This allows for a result within the first 24 to 48 h, facilitating the selection of suitable antibiotic treatment for patients with positive blood cultures [21, 26]. Consequently, incorporating AI in clinical microbiology laboratories can assist in choosing appropriate antibiotic treatment regimens, a critical factor in achieving high cure rates for various infectious diseases [21, 26].

ML research in medicine has rapidly expanded, which could greatly help the healthcare providers in the emergency department (ED) as they face challenging difficulties from the rising burden of diseases, greater demand for time and health services, higher societal expectations, and increasing health expenditures [27]. Emergency department providers understand that integrating AI into their work processes is necessary for solving these problems by enhancing efficiency, and accuracy, and improving patient outcomes [28, 29]. Additionally, there may be a chance for algorithm support and automated decision-making to optimize ED flow measurements and resource allocation [30]. AI algorithms can analyze patient data to assist with triaging patients based on urgency; this helps prioritize high-risk cases, reducing waiting times and improving patient flow [31]. Introducing a reliable symptom assessment tool can rule out other causes of illness to reduce the number of unnecessary visits to the ED. A series of AI-enabled machines can directly question the patient, and a sufficient explanation is provided at the end to ensure appropriate assessment and plan.

Moreover, AI-powered decision support systems can provide real-time suggestions to healthcare providers, aiding diagnosis, and treatment decisions. Patients are evaluated in the ED with little information, and physicians frequently must weigh probabilities when risk stratifying and making decisions. Faster clinical data interpretation is crucial in ED to classify the seriousness of the situation and the need for immediate intervention. The risk of misdiagnosing patients is one of the most critical problems affecting medical practitioners and healthcare systems. Diagnostic mistakes in the healthcare sector can be expensive and fatal. A study found that diagnostic errors, particularly in patients who visit the ED, directly contribute to a greater mortality rate and a more extended hospital stay [32]. Fortunately, AI can assist in the early detection of patients with life-threatening diseases and promptly alert clinicians so the patients can receive immediate attention. Lastly, AI can help optimize health care sources in the ED by predicting patient demand, optimizing therapy selection (medication, dose, route of administration, and urgency of intervention), and suggesting emergency department length of stay. By analyzing patient-specific data, AI systems can offer insights into optimal therapy selection, improving efficiency and reducing overcrowding.

### AI in genomic medicine

**Fig. 2:**
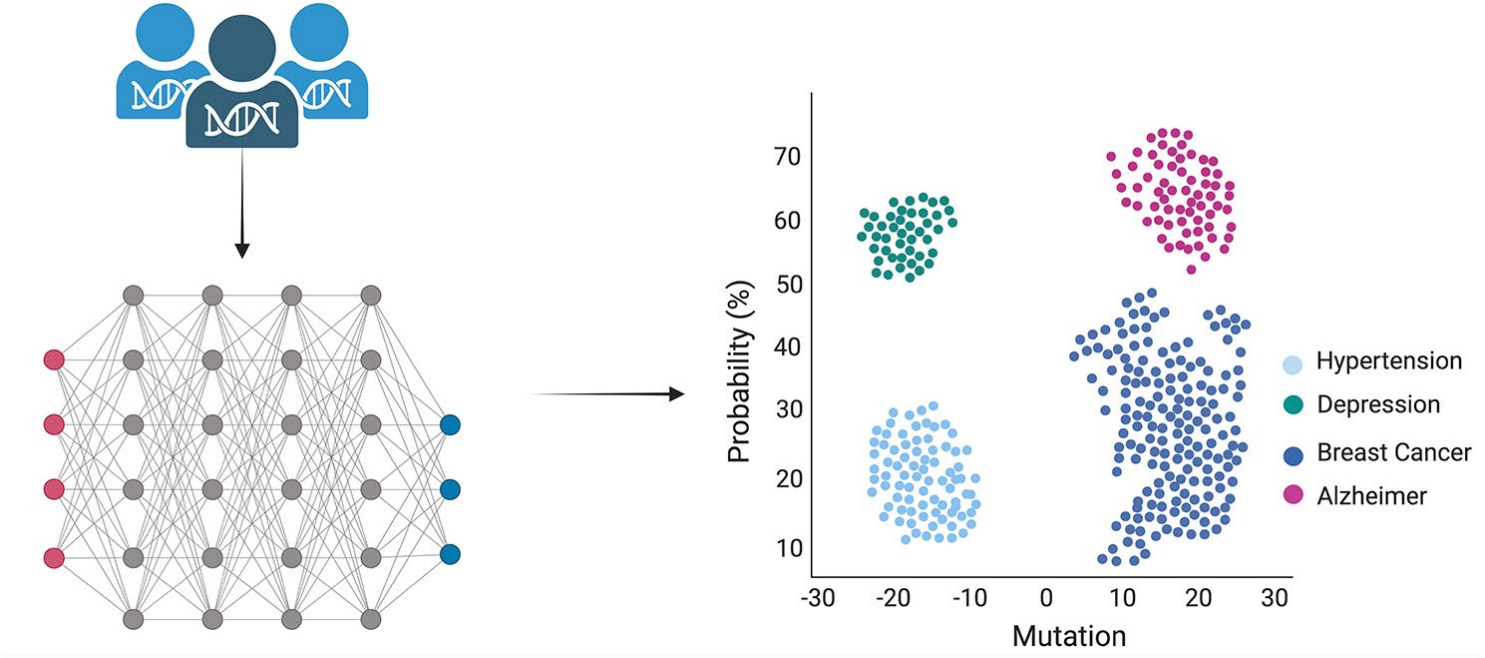
Schematic representation of the process starting with the extraction of DNA/RNA, followed by sequencing. The subsequent genotypic alignment is performed using neural networks and deep learning. Probability calculations are achieved through applying statistical methods and M: The graph’s Y-axis denotes the probability (expressed in percentage) of a particular type of disease (hypertension, depression, breast cancer, and Alzheimer’s disease), while the X-axis signifies the count of gene mutations. Negative numbers indicate gene deletions, whereas positive values represent gene additions or nucleic acid mutations

The fusion of AI and genotype analysis holds immense promise in the realms of disease surveillance, prediction, and personalized medicine [33]. When applied to large populations, AI can effectively monitor for emerging disease threats (such as COVID-19), while genomic data can provide valuable insights into genetic markers associated with increased susceptibility to specific diseases [34] By training ML algorithms to identify these markers in real-time data, we can facilitate the early detection of potential outbreaks. Moreover, the use of genotype data can aid in refining disease risk predictions, as ML algorithms can recognize complex patterns of genetic variations linked with disease susceptibility that might elude traditional statistical methods as summarized in Fig. 2 [35, 36]. The prediction of phenotypes, or observable characteristics shaped by genes and environmental factors, also becomes possible with this combination.

ML algorithms make it feasible to predict a spectrum of phenotypes ranging from simple traits like eye color to more intricate ones like the response to certain medications or disease susceptibility. A specific area where AI and ML have demonstrated significant efficacy is the identification of genetic variants associated with distinctive traits or pathologies. Examining extensive genomic datasets allows these techniques to detect intricate patterns often elusive to manual analysis. For instance, a groundbreaking study employed a deep neural network to identify genetic variants associated with autism spectrum disorder (ASD), successfully predicting ASD status by relying solely on genomic data [37]. In the field of oncology, categorizing cancers into clinically relevant molecular subtypes can be accomplished using transcriptomic profiling. Such molecular classifications, first developed for breast cancer and later extended to other cancers like colorectal, ovarian, and sarcomas, hold substantial implications for diagnosis, prognosis, and treatment selection [38, 39]. Traditional computational methods for subtyping cancers, such as support vector machines (SVMs) or k-nearest neighbors, are susceptible to errors due to batch effects and may only focus on a small set of signature genes, thus neglecting vital biological information [40].

The advent of high-throughput genomic sequencing technologies, combined with advancements in AI and ML, has laid a strong foundation for accelerating personalized medicine and drug discovery [41]. Despite being a treasure trove of valuable insights, the complex nature of extensive genomic data presents substantial obstacles to its interpretation. The field of drug discovery has dramatically benefited from the application of AI and ML. The simultaneous analysis of extensive genomic data and other clinical parameters, such as drug efficacy or adverse effects, facilitates the identification of novel therapeutic targets or the repurposing of existing drugs for new applications [42–46]. One of the prevalent challenges in drug development is non-clinical toxicity, which leads to a significant percentage of drug failures during clinical trials. However, the rise of computational modeling is opening up the feasibility of predicting drug toxicity, which can be instrumental in improving the drug development process [46]. This capability is particularly vital for addressing common types of drug toxicity, such as cardiotoxicity and hepatotoxicity, which often lead to post-market withdrawal of drugs.

## AI assistance in treatment

### Precision medicine and clinical decision support

Personalized treatment, also known as precision medicine or personalized medicine, is an approach that tailors medical care to individual patients based on their unique characteristics, such as genetics, environment, lifestyle, and biomarkers [47]. This individualized approach aims to improve patient outcomes by providing targeted interventions that are more effective, efficient, and safe. AI has emerged as a valuable tool in advancing personalized treatment, offering the potential to analyze complex datasets, predict outcomes, and optimize treatment strategies [47, 48]. Personalized treatment represents a pioneering field that demonstrates the potential of precision medicine on a large scale [49]. Nevertheless, the ability to provide real-time recommendations relies on the advancement of ML algorithms capable of predicting patients who may require specific medications based on genomic information. The key to tailoring medications and dosages to patients lies in the pre-emptive genotyping of patients prior to the actual need for such information [49, 50].

The potential applications of AI in assisting clinicians with treatment decisions, particularly in predicting therapy response, have gained recognition [49]. A study conducted by Huang et al. where authors utilized patients’ gene expression data for training a support ML, successfully predicted the response to chemotherapy [51]. In this study, the authors included 175 cancer patients incorporating their gene-expression profiles to predict the patients’ responses to various standard-of-care chemotherapies. Notably, the research showed encouraging outcomes, achieving a prediction accuracy of over 80% across multiple drugs. These findings demonstrate the promising role of AI in treatment response prediction. In another study performed by Sheu et al., the authors aimed to predict the response to different classes of antidepressants using electronic health records (EHR) of 17,556 patients and AI [52]. The AI models considered features predictive of treatment selection to minimize confounding factors and showed good prediction performance. The study demonstrated that antidepressant response could be accurately predicted using real-world EHR data with AI modeling, suggesting the potential for developing clinical decision support systems for more effective treatment selection. While considerable progress has been made in leveraging AI techniques and genomics to forecast treatment outcomes, it is essential to conduct further prospective and retrospective clinical research and studies [47, 50]. These endeavors are necessary for generating the comprehensive data required to train the algorithms effectively, ensure their reliability in real-world settings, and further develop AI-based clinical decision tools.

### Dose optimization and therapeutic drug monitoring

AI plays a crucial role in dose optimization and adverse drug event prediction, offering significant benefits in enhancing patient safety and improving treatment outcomes [53]. By leveraging AI algorithms, healthcare providers can optimize medication dosages tailored to individual patients and predict potential adverse drug events, thereby reducing risks and improving patient care. In a study that aimed to develop an AI-based prediction model for prothrombin time international normalized ratio (PT/INR) and a decision support system for warfarin maintenance dose optimization [54] The authors analyzed data from 19,719 inpatients across three institutions, and the algorithm outperformed expert physicians with significant differences in predicting future PT/INRs and the generated individualized warfarin dose was reliable.

On the contrary, a novel dose optimization system—CURATE.AI—is an AI-derived platform for dynamically optimizing chemotherapy doses based on individual patient data [55]. A study was conducted to validate this system as an open-label, prospective trial in patients with advanced solid tumors treated with three different chemotherapy regimens. CURATE.AI generated personalized doses for subsequent cycles based on the correlation between chemotherapy dose variation and tumor marker readouts. The integration of CURATE.AI into the clinical workflow showed successful incorporation and potential benefits in terms of reducing chemotherapy dose and improving patient response rates and durations compared to the standard of care. These findings support the need for prospective validation through randomized clinical trials and indicate the potential of AI in optimizing chemotherapy dosing and lowering the risk of adverse drug events.

Therapeutic drug monitoring (TDM) is a process used to optimize drug dosing in individual patients. It is predominantly utilized for drugs with a narrow therapeutic index to avoid both underdosing insufficiently medicating as well as toxic levels. TDM aims to ensure that patients receive the right drug, at the right dose, at the right time, to achieve the desired therapeutic outcome while minimizing adverse effects [56]. The use of AI in TDM has the potential to revolutionize how drugs are monitored and prescribed. AI algorithms can be trained to predict an individual’s response to a given drug based on their genetic makeup, medical history, and other factors. This personalized approach to drug therapy can lead to more effective treatments and better patient outcomes [57, 58].

One example of AI in TDM is using ML algorithms to predict drug-drug interactions. By analyzing large datasets of patient data, these algorithms can identify potential drug interactions. This can help to reduce the risk of adverse drug reactions, and cost and improve patient outcomes [59]. Another application of AI in TDM using predictive analytics to identify patients at high risk of developing adverse drug reactions. By analyzing patient data and identifying potential risk factors, healthcare providers can take proactive steps to prevent adverse events before they occur [60]. Overall, the use of AI in TDM has the potential to improve patient outcomes, reduce healthcare costs, and enhance the accuracy and efficiency of drug dosing. As this technology continues to evolve, AI will likely play an increasingly important role in the field of TDM.

## AI assistance in population health management

### Predictive analytics and risk assessment

**Fig. 3:**
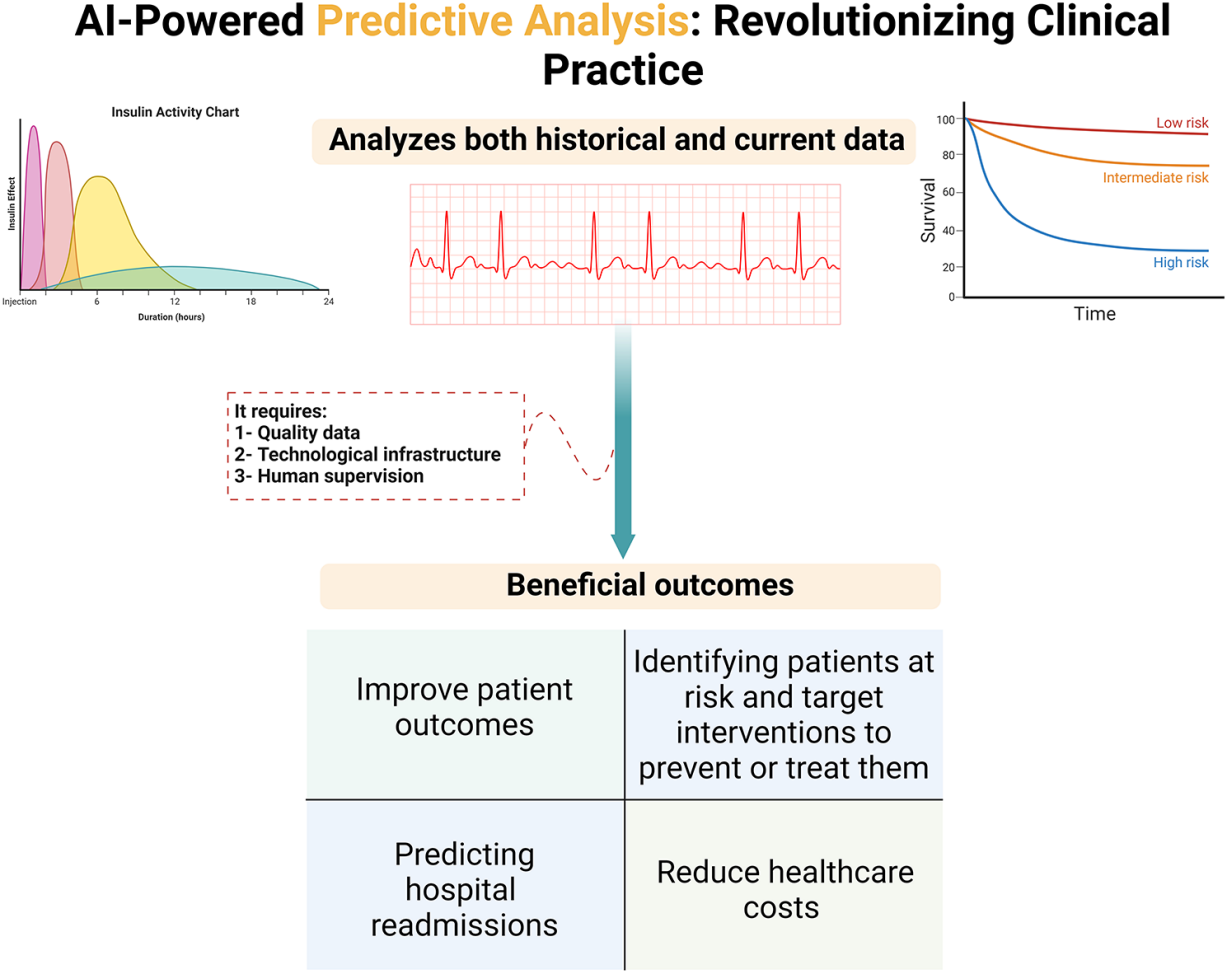
Unlocking the Power of Patient Data with AI-Driven Predictive Analytics

Population health management increasingly uses predictive analytics to identify and guide health initiatives. In data analytics, predictive analytics is a discipline that significantly utilizes modeling, data mining, AI, and ML. In order to anticipate the future, it analyzes historical and current data [61, 62]. ML algorithms and other technologies are used to analyze data and develop predictive models to improve patient outcomes and reduce costs. One area where predictive analytics can be instrumental is in identifying patients at risk of developing chronic diseases such as endocrine or cardiac diseases. By analyzing data such as medical history, demographics, and lifestyle factors, predictive models can identify patients at higher risk of developing these conditions and target interventions to prevent or treat them [61]. Predicting hospital readmissions is another area where predictive analytics can be applied. By analyzing patient demographics, medical history, and social health factors, predictive models can identify patients at higher risk of hospital readmissions and target interventions to prevent readmissions (Fig. 3) [62–64]; this can help reduce healthcare costs and improve patient outcomes which is the reason behind launching new companies such as “Reveal ®” [65].

AI can be used to optimize healthcare by improving the accuracy and efficiency of predictive models. AI algorithms can analyze large amounts of data and identify patterns and relationships that may not be obvious to human analysts; this can help improve the accuracy of predictive models and ensure that patients receive the most appropriate interventions. AI can also automate specific public health management tasks, such as patient outreach and care coordination [61, 62]. Which can help reduce healthcare costs and improve patient outcomes by ensuring patients receive timely and appropriate care. However, it is pivotal to note that the success of predictive analytics in public health management depends on the quality of data and the technological infrastructure used to develop and implement predictive models. In addition, human supervision is vital to ensure the appropriateness and effectiveness of interventions for at-risk patients. In summary, predictive analytics plays an increasingly important role in population health. Using ML algorithms and other technologies, healthcare organizations can develop predictive models that identify patients at risk for chronic disease or readmission to the hospital [61–64].

Furthermore, AI is needed to address these challenges regarding vaccine production and supply chain bottlenecks. Testing algorithms on real-time vaccine supply chains can be challenging. To overcome this, investing in research and development is essential to create robust algorithms that can accurately predict and optimize vaccine supply chains. Edge analytics can also detect anomalies and predict Disease X events and associated risks to the healthcare system [66].

From a Saudi perspective, Sehaa, a big data analytics tool in Saudi Arabia, uses Twitter data to detect diseases, and it found that dermal diseases, heart diseases, hypertension, cancer, and diabetes are the top five diseases in the country [67]. Riyadh has the highest awareness-to-afflicted ratio for six of the fourteen diseases detected, while Taif is the healthiest city with the lowest number of disease cases and a high number of awareness activities. These findings highlight the potential of predictive analytics in population health management and the need for targeted interventions to prevent and treat chronic diseases in Saudi Arabia [67]. AI can optimize health care by improving the accuracy and efficiency of predictive models and automating certain tasks in population health management [62]. However, successfully implementing predictive analytics requires high-quality data, advanced technology, and human oversight to ensure appropriate and effective interventions for patients.

### Establishment of working groups, guidelines, and frameworks

AI is transforming how guidelines are established in various fields. In healthcare, guidelines usually take much time, from establishing the knowledge gap that needs to be fulfilled to publishing and disseminating these guidelines. AI can help identify newly published data based on data from clinical trials and real-world patient outcomes within the same area of interest which can then facilitate the first stage of mining information. Then, under the supervision of scientists and experts in the field, AI algorithms can analyze vast amounts of data to identify patterns and trends that can inform the development of evidence-based guidelines in real-time, which allows for a fast exchange of information with essential supervision clinicians for its clinical and ethical implications [68–73].

Several professional organizations have developed frameworks for addressing concerns unique to developing, reporting, and validating AI in medicine [69–73]. Instead of focusing on the clinical application of AI, these frameworks are more concerned with educating the technological creators of AI by providing instructions on encouraging transparency in the design and reporting of AI algorithms [69]. Additionally, regulatory regulation of AI is still in its infancy. The US Food and Drug Administration (FDA) is now developing guidelines on critically assessing real-world applications of AI in medicine while publishing a framework to guide the role of AI and ML in software as medical devices [74]. The European Commission has spearheaded a multidisciplinary effort to improve the credibility of AI [75], and the European Medicines Agency (EMA) has deemed the regulation of AI a strategic priority [76]. These legislative efforts are meant to shape the healthcare future to be better equipped to be a technology-driven sector. Overall, the role of AI in establishing guidelines is to provide data-driven insights and recommendations based on vast amounts of information, which can lead to more efficient and effective decision-making, better outcomes, and reduced costs. However, it is crucial to ensure that AI-based guidelines are transparent, fair, unbiased, and informed by human expertise and ethical considerations [68].

### AI in drug information and consultation

AI would propose a new support system to assist practical decision-making tools for healthcare providers. In recent years, healthcare institutions have provided a greater leveraging capacity of utilizing automation-enabled technologies to boost workflow effectiveness and reduce costs while promoting patient safety, accuracy, and efficiency [77]. By introducing advanced technologies like NLP, ML, and data analytics, AI can significantly provide real-time, accurate, and up-to-date information for practitioners at the hospital. According to the McKinsey Global Institute, ML and AI in the pharmaceutical sector have the potential to contribute approximately $100 billion annually to the US healthcare system [78]. Researchers claim that these technologies enhance decision-making, maximize creativity, increase the effectiveness of research and clinical trials, and produce new tools that benefit healthcare providers, patients, insurers, and regulators [78]. AI enables quick and comprehensive retrieval of drug-related information from different resources through its ability to analyze the current medical literature, drug databases, and clinical guidelines to provide accurate and evidence-based decisions for healthcare providers. Using automated response systems, AI-powered virtual assistants can handle common questions and provide detailed medical information to healthcare providers [79]. AI-powered chatbots help reduce the workload on healthcare providers, allowing them to focus on more complicated cases that require their expertise. Also, AI algorithms can generate specific recommendations for individual patients, considering factors like health conditions, past medical and medication history, and social/lifestyle preferences, allowing healthcare professionals to optimize medication choices and dosages [80, 81].

## AI-powered patient care

### AI virtual healthcare assistance

With continuously increasing demands of health care services and limited resources worldwide, finding solutions to overcome these challenges is essential [82]. Virtual health assistants are a new and innovative technology transforming the healthcare industry to support healthcare professionals. It is designed to simulate human conversation to offer personalized patient care based on input from the patient [83]. These digital assistants use AI-powered applications, chatbots, sounds, and interfaces. Virtual assistants can help patients with tasks such as identifying the underlying problem based on the patient’s symptoms, providing medical advice, reminding patients to take their medications, scheduling doctor appointments, and monitoring vital signs. In addition, digital assistants can collect information daily regarding patients’ health and forward the reports to the assigned physician. By taking off some of these responsibilities from human healthcare providers, virtual assistants can help to reduce their workload and improve patient outcomes.

Furthermore, these tools can always be available, making it easier for patients to access healthcare when needed [84]. Another medical service that an AI-driven phone application can provide is triaging patients and finding out how urgent their problem is, based on the entered symptoms into the app. The National Health Service (NHS) has tested this app in north London, and now about 1.2 million people are using this AI chatbot to answer their questions instead of calling the NHS non-emergency number [85]. In addition, introducing intelligent speakers into the market has a significant benefit in the lives of elderly and chronically ill patients who are unable to use smartphone apps efficiently [86]. Overall, virtual health assistants have the potential to significantly improve the quality, efficiency, and cost of healthcare delivery while also increasing patient engagement and providing a better experience for them.

### AI mental health support

AI has the potential to revolutionize mental health support by providing personalized and accessible care to individuals [87, 88]. Several studies showed the effectiveness and accessibility of using Web-based or Internet-based cognitive-behavioral therapy (CBT) as a psychotherapeutic intervention [89, 90]. Even though psychiatric practitioners rely on direct interaction and behavioral observation of the patient in clinical practice compared to other practitioners, AI-powered tools can supplement their work in several ways. AI-powered mental health applications can assist in the early detection and diagnosis of mental health conditions, as well as provide tailored treatment and support [88–91] These applications can also offer round-the-clock support, reducing the need for in-person appointments and wait times. Furthermore, these digital tools can be used to monitor patient progress and medication adherence, providing valuable insights into treatments’ effectiveness [88].

The current published studies addressing the applicability of AI in mental health concluded that depression is the most commonly investigated mental disorder [88]. Moreover, AI-powered apps prove their benefits in patients with substance use disorder. A recent study evaluated the utility of a mental health digital app called Woebot in patients with substance use disorders. This study found that using Woebot was significantly associated with improved substance use, cravings, depression, and anxiety [92]. While AI-powered mental health diagnosis holds promise, some significant limitations must be addressed. One of the main limitations is the risk of bias in the data and algorithms used in AI-powered diagnosis. If the data used to train AI algorithms does not represent diverse populations, it can lead to biased and inaccurate results. Additionally, AI-powered diagnosis may not take into account the complexity of mental health conditions, which can present differently in different people. Finally, there is a risk that AI-powered diagnosis may lead to a lack of personalization and empathy in mental health care, which is an important aspect of successful treatment [93]. Therefore, while AI-powered diagnosis can be a valuable tool in mental health care, it should be used as a supplement to, rather than a replacement for, professional diagnosis and treatment.

### AI in enhancing patient education and mitigating healthcare provider burnout

One of the emerging applications of AI is patient education [94]. AI-powered chatbots are being implemented in various healthcare contexts, such as diet recommendations [95, 96], smoking cessation, and cognitive-behavioral therapy [97]. Patient education is integral to healthcare, as it enables individuals to understand their medical diagnosis, treatment options, and preventative measures [98]. Informed patients are more likely to adhere to their treatment regimens and achieve better health outcomes [99]. AI has the potential to play a significant role in patient education by providing personalized and interactive information and guidance to patients and their caregivers [100]. For example, in patients with prostate cancer, introducing a prostate cancer communication assistant (PROSCA) chatbot offered a clear to moderate increase in participants’ knowledge about prostate cancer [101]. Researchers found that ChatGPT, an AI Chatbot founded by OpenAI, can help patients with diabetes understand their diagnosis and treatment options, monitor their symptoms and adherence, provide feedback and encouragement, and answer their questions [102]. AI technology can also be applied to rewrite patient education materials into different reading levels. This suggests that AI can empower patients to take greater control of their health by ensuring that patients can understand their diagnosis, treatment options, and self-care instructions [103]. However, there are also some challenges and limitations that need to be addressed, such as ensuring the accuracy, reliability, and transparency of the information provided by AI, respecting the privacy and confidentiality of the patients’ data, and maintaining a human touch and empathy in the communication [104]. The use of AI in patient education is still in its early stages, but it has the potential to revolutionize the way that patients learn about their health. As AI technology continues to develop, we can expect to see even more innovative and effective ways to use AI to educate patients.

### Are individuals more inclined towards AI than human healthcare providers

Public perception of the benefits and risks of AI in healthcare systems is a crucial factor in determining its adoption and integration. People’s feelings about AI replacing or augmenting human healthcare practitioners, its role in educating and empowering patients, and its impact on the quality and efficiency of care, as well as on the well-being of healthcare workers, are all important considerations. In medicine, patients often trust medical staff unconditionally and believe that their illness will be cured due to a medical phenomenon known as the placebo effect. In other words, patient-physician trust is vital in improving patient care and the effectiveness of their treatment [105]. For the relationship between patients and an AI-based healthcare delivery system to succeed, building a relationship based on trust is imperative [106].

Research on whether people prefer AI over healthcare practitioners has shown mixed results depending on the context, type of AI system, and participants’ characteristics [107, 108]. Some surveys have indicated that people are generally willing to use or interact with AI for health-related purposes such as diagnosis, treatment, monitoring, or decision support [108–110]. However, other studies have suggested that people still prefer human healthcare practitioners over AI, especially for complex or sensitive issues such as mental health, chronic diseases, or end-of-life care [108, 111]. In a US-based study, 60% of participants expressed discomfort with providers relying on AI for their medical care. However, the same study found that 80% of Americans would be willing to use AI-powered tools to help manage their health [109]. In another survey, responders’ comfort with AI varied based on clinical application, and most patients felt that AI would improve their healthcare, which suggests that people are generally willing to use AI for healthcare-related purposes and that patient education, concerns, and comfort levels should be accounted for when planning for integration of AI [110]. Moreover, people’s trust and acceptance of AI may vary depending on their age, gender, education level, cultural background, and previous experience with technology [111, 112].

## Future directions and considerations for clinical implementation

### Obstacles and solutions

AI has the potential to revolutionize clinical practice, but several challenges must be addressed to realize its full potential. Among these challenges is the lack of quality medical data, which can lead to inaccurate outcomes. Data privacy, availability, and security are also potential limitations to applying AI in clinical practice. Additionally, determining relevant clinical metrics and selecting an appropriate methodology is crucial to achieving the desired outcomes. Human contribution to the design and application of AI tools is subject to bias and could be amplified by AI if not closely monitored [113]. The AI-generated data and/or analysis could be realistic and convincing; however, hallucination could also be a major issue which is the tendency to fabricate and create false information that cannot be supported by existing evidence [114]. This can be particularly problematic regarding sensitive areas such as patient care. Thus, the development of AI tools has implications for current health professions education, highlighting the necessity of recognizing human fallibility in areas including clinical reasoning and evidence-based medicine [115]. Finally, human expertise and involvement are essential to ensure the appropriate and practical application of AI to meet clinical needs and the lack of this expertise could be a drawback for the practical application of AI.

Addressing these challenges and providing constructive solutions will require a multidisciplinary approach, innovative data annotation methods, and the development of more rigorous AI techniques and models. Creating practical, usable, and successfully implemented technology would be possible by ensuring appropriate cooperation between computer scientists and healthcare providers. By merging current best practices for ethical inclusivity, software development, implementation science, and human-computer interaction, the AI community will have the opportunity to create an integrated best practice framework for implementation and maintenance [116]. Additionally, a collaboration between multiple health care settings is required to share data and ensure its quality, as well as verify analyzed outcomes which will be critical to the success of AI in clinical practice. Another suggestion is to provide appropriate training and education that starts from the undergraduate level of all the health care practitioners and proceeds to the continuous development and improvement for the practitioners working in the current practice to ensure the proper adaptation which provides the best patient care and avoid any legal or ethical issues or misinterpretation of the outcomes without verifying the results [117]. Medical schools are encouraged to incorporate AI-related topics into their medical curricula. A study conducted among radiology residents showed that 86% of students agreed that AI would change and improve their practice, and up to 71% felt that AI should be taught at medical schools for better understanding and application [118]. This integration ensures that future healthcare professionals receive foundational knowledge about AI and its applications from the early stages of their education.

### Legal, ethical, and risk associated with AI in healthcare system

Converting AI and big data into secure and efficient practical applications, services, and procedures in healthcare involves significant costs and risks. Consequently, safeguarding the commercial interests of AI and data-driven healthcare technologies has emerged as an increasingly crucial subject [119]. In the past, only medical professionals could measure vital signs such as blood pressure, glucose levels, and heart rate [48]. However, contemporary mobile applications now enable the continuous collection of such information. Nevertheless, addressing the ethical risks associated with AI implementation is imperative, particularly concerning data privacy and confidentiality violations, informed consent, and patient autonomy [48, 119]. Given the prominence of big data and AI in healthcare and precision medicine, robust data protection legislation becomes paramount to safeguarding individual privacy. Countries around the world have introduced laws to protect the privacy of their citizens, such as the Health Insurance Portability and Accountability Act (HIPAA) in the United States and the General Data Protection Regulation (GDPR) in Europe [120, 121]. While HIPAA protects only relevant health information produced by covered entities, the GDPR has implemented extensive data protection law within the EU, creating a significant global shift in data protection [120, 121].

One of the major causes that can compromise patient data, disrupt critical healthcare operations, and jeopardize patient safety with the use of AI in the healthcare system is increased cyberattacks [66, 122]. Predictive algorithms can be employed to detect and prevent these cyber threats. To safeguard data privacy and maintain system integrity, it’s essential to deeply investigate cybersecurity and the cyber risk landscape of healthcare systems [66, 123]. By implementing a variety of robust AI algorithms, the risk associated with relying on a singular solution can be mitigated [66, 123]. While data privacy and security breaches are challenges associated with AI in healthcare [122], it offers significant advantages such as task streamlining, enhanced efficiency, time and resource savings, research support, and reduced physician stress [122]. In the context of ethical considerations, an epistemological framework for ethical assessment has been proposed to prioritize ethical awareness, transparency, and accountability when evaluating digital technology’s impact on healthcare supply chain participants [123, 124].

## Conclusion

The integration of AI in healthcare has immense potential to revolutionize patient care and outcomes. AI-driven predictive analytics can enhance the accuracy, efficiency, and cost-effectiveness of disease diagnosis and clinical laboratory testing. Additionally, AI can aid in population health management and guideline establishment, providing real-time, accurate information and optimizing medication choices. Integrating AI in virtual health and mental health support has shown promise in improving patient care. However, it is important to address limitations such as bias and lack of personalization to ensure equitable and effective use of AI.

Several measures must be taken to ensure responsible and effective implementation of AI in healthcare.

Firstly, comprehensive cybersecurity strategies and robust security measures should be developed and implemented to protect patient data and critical healthcare operations. Collaboration between healthcare organizations, AI researchers, and regulatory bodies is crucial to establishing guidelines and standards for AI algorithms and their use in clinical decision-making. Investment in research and development is also necessary to advance AI technologies tailored to address healthcare challenges.

AI algorithms can continuously examine factors such as population demographics, disease prevalence, and geographical distribution. This can identify patients at a higher risk of certain conditions, aiding in prevention or treatment. Edge analytics can also detect irregularities and predict potential healthcare events, ensuring that resources like vaccines are available where most needed.

Public perception of AI in healthcare varies, with individuals expressing willingness to use AI for health purposes while still preferring human practitioners in complex issues. Trust-building and patient education are crucial for the successful integration of AI in healthcare practice. Overcoming challenges like data quality, privacy, bias, and the need for human expertise is essential for responsible and effective AI integration.

Collaboration among stakeholders is vital for robust AI systems, ethical guidelines, and patient and provider trust. Continued research, innovation, and interdisciplinary collaboration are important to unlock the full potential of AI in healthcare. With successful integration, AI is anticipated to revolutionize healthcare, leading to improved patient outcomes, enhanced efficiency, and better access to personalized treatment and quality care.

## Data Availability

Not applicable.

## Abbreviations

AI: Artificial Intelligence
ASD: Autism Spectrum Disorder
CCN: Convolutional Neural Networks
DIHM: Digital in-line holographic microscopy
GDPR: General Data Protection Regulation
EHR: Electronic Health Record
HIPAA: Health Insurance Portability and Accountability Act
LLMs: Large Language Models
ML: Machine Learning
NLP: Natural Language Processing
PROSCA: Prostate cancer communication assistant
PT/INR: Prothrombin Time / International Normalized Ratio
SVMs: Support vector Machines
TDM: Therapeutic drug monitoring
